# Optical Genome Mapping Reveals and Characterizes Recurrent Aberrations and New Fusion Genes in Adult ALL

**DOI:** 10.3390/genes14030686

**Published:** 2023-03-09

**Authors:** Lisa-Marie Vieler, Verena Nilius-Eliliwi, Roland Schroers, Deepak Ben Vangala, Huu Phuc Nguyen, Wanda Maria Gerding

**Affiliations:** 1 Department of Human Genetics, Ruhr-University Bochum, Universitätsstr. 150, 44801 Bochum, Germany; 2 Center for Hemato-Oncological Diseases, University Hospital Knappschaftskrankenhaus Bochum, In der Schornau 23-25, 44892 Bochum, Germany

**Keywords:** optical genome mapping (OGM), adult acute lymphoblastic leukemia (ALL), CDKN2A/B deletion, ring chromosome, isodicentric chromosome

## Abstract

(1) Background: In acute lymphoblastic leukemia (ALL) the genetic characterization remains challenging. Due to the genetic heterogeneity of mutations in adult patients, only a small proportion of aberrations can be analyzed with standard routine diagnostics. Optical genome mapping (OGM) has recently opened up new possibilities for the characterization of structural variants on a genome-wide level, thus enabling simultaneous analysis for a broad spectrum of genetic aberrations. (2) Methods: 11 adult ALL patients were examined using OGM. (3) Results: Genetic results obtained by karyotyping and FISH were confirmed by OGM for all patients. Karyotype was redefined, and additional genetic information was obtained in 82% (9/11) of samples by OGM, previously not diagnosed by standard of care. Besides gross-structural chromosome rearrangements, e.g., ring chromosome 9 and putative isodicentric chromosome 8q, deletions in *CDKN2A/2B* were detected in 7/11 patients, defining an approx. 20 kb minimum region of overlap, including an alternative exon 1 of the *CDKN2A* gene. The results further confirm recurrent ALL aberrations (e.g., *PAX5*, *ETV6*, *VPREB1*, *IKZF1*). (4) Conclusions: Genome-wide OGM analysis enables a broad genetic characterization in adult ALL patients in one single workup compared to standard clinical testing, facilitating a detailed genetic diagnosis, risk-stratification, and target-directed treatment strategies.

## 1. Introduction

Acute lymphoblastic leukemia (ALL) results from malignant transformation during the early stages of lymphocytic development [1]. T- and B-cells account for 75% and 25% of cases, respectively [2]. ALL incidence peaks in children below 5 years (incidence 5,3/100.000) and in adults >80 years (incidence 2,3/100.000) [3]. ALL survival rates in adults are subtype-dependent, ranging from 35 to 50%, but are about 80% for pediatric ALL patients [4]. The classification of ALL by the World Health Organization (WHO) is primarily based on genetic alterations that can be found in malignant precursor cells. Although there are recurrent genetic events known in T-ALL [5,6], the classification of subgroups defined by genetic variants is only given for B-ALL. In the novel WHO 2022 classification, the increased number of genetic subtypes emphasizes the importance of genetic testing on initial diagnosis and follow-up [7]. Several B-ALL subclasses are based on recurrent, distinct genetic features, including the well-studied Philadelphia chromosome t(9;22)(q34;q11) resulting in the *BCR::ABL1* gene fusion or the recurrent *ETV6::RUNX1* fusion gene [8,9]. However, a category “not otherwise specified” still exists for B-ALL, and the frequency of genetic subtypes strongly varies with age. For example, Philadelphia chromosome-positive (Ph+) ALL is common in adults (40–50%), while it only affects around 5% of pediatric cases. In contrast, hyperdiploid karyotypes or *ETV6::RUNX1* positive cases are often found in children (both around 25% in childhood ALL) and are associated with a better prognosis compared to frequent aberrations in adults [10]. A local risk stratification from the German Multicenter Study Group on Adult Acute Lymphoblastic Leukemia (GMALL) is available, but only a few genetic aberrations are considered: translocations t(9;22) *BCR::ABL1* and t(4;11) *KMT2A::AFF1* are included as adverse risk factors. In general, limited information regarding classification and risk stratification is currently available for ALL based on genetic alterations, in particular for adult patients, and needs to be extended. 

ALL diagnostics in standard of care are carried out using different methodologies: karyotyping allows the characterization of large structural aberrations with the limitation of its maximal resolution in the range of 5–10 megabases (Mb). Moreover, it depends on the quality of material and preparation of metaphase chromosomes. FISH and RT-PCR enable a higher resolution beyond the resolution of karyotyping but require a specific target mutation. In contrast, optical genome mapping (OGM) allows a genome-wide approach with a resolution of up to 500 bp depending on the analysis settings for somatic aberrations or germline variants, respectively. The benefit of OGM analysis was studied in patients with acute myeloid leukemia (AML) before [11,12,13,14,15]: It enables a genome-wide analysis in one single workflow without the necessity to preselect target regions to be analyzed by multiple FISH probes or RT-PCR assays. In addition, OGM analysis was already performed in a few studies examining ALL patients: Lestringant et al. compared OGM with a combination of conventional methods in 7 pediatric and 3 adult ALL patients. More than 90% of the variants plus additional genetic information were shown to be detected by OGM [16]. Twelve childhood ALL samples were studied by Lühmann et al., where OGM confirmed known aberrations and detected additional results [17]. Rack et al. examined 26 pediatric and 15 adult ALL patients and revealed an increased detection rate and resolution as well as more precise information about breakpoint regions by single workup OGM analysis compared to conventional analyses [18]. Thus, this method has the potential to provide a faster and more accurate diagnosis, especially in adult ALL patients where many genes can be studied in parallel that are not yet part of genetic risk stratification and are rarely analyzed in clinical routine diagnostics. In this study, 11 adult patients (9 B-ALL, 2 T-ALL cases) are examined with the aim to characterize structural variants, evaluate the benefit of OGM compared to standard routine clinical testing, and provide a genome-wide view of specific characteristics in adult ALL genetics. 

## 2. Materials and Methods

### 2.1. Patient Samples

Bone marrow samples were collected from 10 patients with newly diagnosed or relapsed ALL during routine clinical diagnostics at the University Hospital Knappschaftskrankenhaus Bochum. Blood was collected from one patient because the bone marrow tap was dry in this case. Conventional diagnostic analyses, including hematopathology, flow cytometry, and cytology, were performed to confirm the diagnosis. Standard-of-care genetic analyses were obtained from commercial diagnostic laboratories and included karyotyping and RT-PCR-based panel testing. Additional FISH analysis was performed in 1 case. EDTA samples for OGM analysis were frozen at –40 °C within 72 h at the Department for Human Genetics, Ruhr-University Bochum. All patients gave written informed consent for participating in the study, which was approved by the local ethics committee.

### 2.2. High-Molecular Weight DNA Isolation and Labeling

High molecular weight (HMW)-DNA was isolated using the Bionano SP Bone Marrow Aspirate (BMA) DNA Isolation Kit or Bionano SP Blood & Cell Culture DNA Isolation Kit (Bionano Genomics, San Diego, CA, USA), and isolation was performed according to the manufacturer’s protocol. HMW-DNA concentrations from 26 to 100 ng/µL were used for labeling with the Direct Label and Stain (DLS) Kit (including DLE-1 enzyme) according to the Bionano Prep Direct Label and Stain (DLS) Protocol (Bionano Genomics, San Diego, CA, USA). For samples with an HMW-DNA concentration higher than the recommended range, the protocol was modified and DNA was diluted using TE buffer (Table S1). Labeled DNA was protected from light and short-term stored overnight at 20 °C before further analysis.

### 2.3. Optical Genome Mapping and Rare Variant Pipeline

Labeled DNA samples were loaded on a Bionano Genomics Saphyr Chip G2.3 and run and visualized in nanochannels on a Bionano Genomics Saphyr System (Bionano Genomics, San Diego, CA, USA). Basically, DNA molecules are linearized in nanochannels on the chip by electrophoresis and imaged using fluorescence microscopy and OGM software solutions (Bionano Access 1.7.1.1. and Bionano Solve 3.7). A target throughput of 1500 Gb for molecules ≥ 150 kb was set to ensure effective coverage ≥ 300×, enabling a detection threshold of about 5% variant allele fraction for structural variants (SVs) and about 10% for copy number variants (CNVs)/aneuploidies for further analysis using the rare variant pipeline (RVP) [19]. Raw molecule files (BNX) were evaluated using quality metrics (map rate ≥ 70%, label density 14 to 17/100 kb, and average N50 ≥ 230 kb for molecules ≥ 150 kb) according to the manufacturer’s instructions (Table S1). RVP analysis, automatically conducted by the Bionano Genomics software mentioned above, was based on BNX-files with molecule data ≥ 150 kbp that are mapped and annotated to the Genome Reference Consortium human build 37 (GRCh37/hg19) and a canonical gene set (hg19). In addition, an OGM control database containing SV loci from 179 ethnically diverse DLE-1-labeled human genomes with no disease phenotypes provided by the manufacturer was used to filter for potentially pathogenic variants. SVs located in highly variable regions according to the rare variant hg19 DLE-1 SV mask provided by the manufacturer were tagged as “masked” and not analyzed by operators but were still available as output (data are available at https://zenodo.org (accessed on 22 December 2022) [DOI: 10.5281/zenodo.7291345]; please also see the data availability statement below). Results in masked regions were not taken into account for further analysis. Confidence filter settings were set to “recommended” by the manufacturer (0 for insertions and deletions, 0.7 for inversions, −1 for duplications, 0.05 for intrachromosomal fusions, and 0.05 for interchromosomal translocations), and variant annotation settings were set to ≤1% SV occurrence compared to the control database. In order to select aberrations that have potential clinical relevance, we further filtered all confident CNVs and rare SVs using a subset of genes, derived from a custom-made Browser Extensible Data (.BED) file containing known genes and breakpoint regions associated with ALL (Table S2 and ALL Genes File S1). The result output for SVs and CNVs was listed in an annotated structural variants file format (SMAP) and visualized as circos plot diagrams and reference-to-sample alignments in a genome browser by Bionano Access software. 

## 3. Results

### 3.1. Patient Characteristics

Optical genome mapping was performed for 9 patients with B-ALL and 2 patients with T-ALL at initial diagnosis or progressive disease. Age ranged from 18 to 62 years; 5 females and 6 males were examined. A detailed summary of patient characteristics is listed in Table 1.

### 3.2. Optical Genome Mapping Results

Additional genetic information obtained through OGM was obtained in 7/11 (64%) patients, redefined karyotyping results in 2/11 patients, and confirmed previous results of standard clinical routine diagnostics without additional information in 2/11 patients. Overall, all variants detected by karyotyping and FISH were confirmed or redefined by OGM. After data filtering, OGM analysis revealed 101 rare SVs and 27 CNVs in the analyzed patients. The number of clinically relevant SVs ranged from 1 to 11, and for CNVs, from 0 to 8 per patient, 2 patients showed aneuploidies of >3 chromosomes. A detailed overview of the results compared to routine clinical testing is listed in Table S3. 

#### 3.2.1. Gross Structural Rearrangements and Aneuploidies

**Figure 1:**
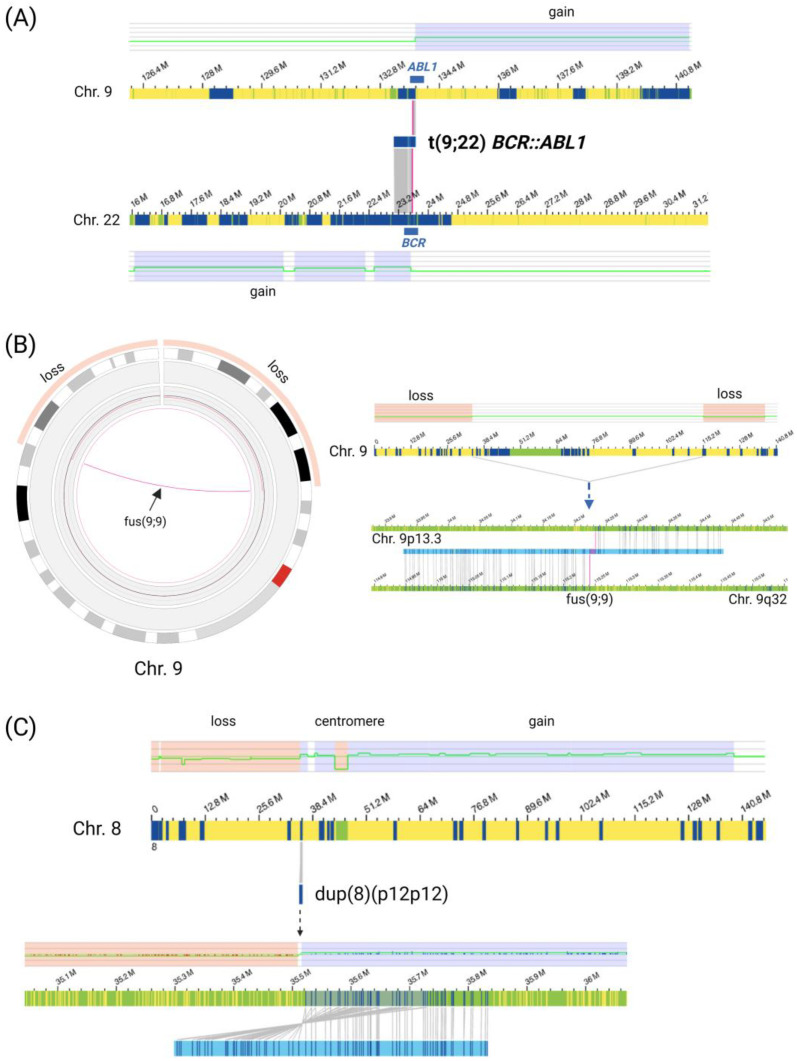
OGM detects different types of gross structural aberrations. (**A**) Additional Philadelphia-like chromosome in patient 2: visualized as interchromosomal translocation (9;22)(q34.12;q11.23) with *BCR::ABL1* fusion in combination with neighboring copy number (CN) gains in close proximity to the translocation breakpoint region. CNV filter settings “no filter” were applied for visualization. (**B**) Presence of a ring chromosome of unknown origin previously detected by karyotyping could be confirmed and redefined by OGM to chromosome 9 material in patient 9. The ring chromosome presents as a translocation SV in combination with distal CN losses in circos plot and SV visualization. (**C**) Putative isodicentric chromosome idic(8q) presenting as CN gains on chromosome 8q and part of 8p in conjunction with an inverted duplication SV on 8p12 and a CN loss in close proximity (patient 4). CNV filter settings were set to “no filter” for visualization. Idic(8q) was not detected by karyotyping. Light blue and light red blocks depict gains and losses of chromosomal material in the respective regions. Blue blocks depict structural variants, green blocks depict masked regions including the centromeric region on chromosome depicted in yellow. Created with BioRender.com.

In the patients analyzed, an interesting variety of structural changes, including an additional short Philadelphia-like chromosome, a ring chromosome 9, and a putative isodicentric chromosome 8q, were detected (Figure 1A–C). One patient (patient 2) showed a hyperdiploid karyotype with 55 chromosomes (trisomies 4, 6, 11, 17, and X, and tetrasomies 14 and 21). Gross structural aberrations ≥ 10 Mb, or aneuploidies based on the approximate resolution of a classical karyogram, were detected in 8/11 patients.

An additional, shortened Philadelphia chromosome consisting of partial chromosome 9 and chromosome 22 material (Figure 1A) was detected in patient 2, confirming the karyotyping results (der(22)t(9;22)(q34;q11)) of the *BCR::ABL1* translocation, detected in one single metaphase. The additional, short Philadelphia chromosome is presented in OGM data as a CN gain next to a *BCR::ABL1* breakpoint region. On chromosome 22, a CN gain spans from next to the *BCR* gene to the centromere (with a fractional CN of about 2.6–2.8 and a corresponding variant allele frequency (VAF) ranging from 0.31–0.41), covering cytobands 22q11.1q11.22 and partially 22q11.23 up to the *BCR*-gene. On chromosome 9, the CN gain (with a fractional CN of around 2.8 and a corresponding VAF of approx. 0.43) spans from the *ABL1* gene on 9q34.13 to the telomeric region of chromosome 9q. OGM output VAF of this aberration corresponds to 15–20% of cells in the sample material and thus might indicate that the detected single metaphase in karyotyping was probably underestimating the proportion of affected cells. Further aberrations in the same sample were present with a higher VAF, suggesting the presence of different malignant clones. In addition, the origin of two marker chromosomes could be resolved: one additional chromosome X, resulting in a trisomy X, and an additional chromosome 21, adding up to a tetrasomy 21. 

A ring chromosome from an unknown origin in combination with a loss of chromosome 9 was identified by karyotyping in patient 9; this result could be confirmed and redefined by OGM analysis: gross losses of about 34 Mbp of the 9p terminal region and 21 Mbp on distal 9q were detected as CNVs. At the borders of these CN losses, SV analysis revealed breakpoints of an intrachromosomal fusion of chromosome 9p and 9q. This structural variant reveals a ring formation of a derivative chromosome 9 (Figure 1B). In this patient, OGM also redefined the karyotype, resolving another gross rearrangement between chromosomes 5 and 11, and possibly 8 and 13. These translocations were detected at low VAF, ranging from 0.16–0.21 for t(5;11)(q35;q23) and t(5;11)(q35;q24) to 0.02–0.04 for t(8;13)(q24.22;q21.32). Because of the low VAF values for the t(8;13), this result has to be interpreted with caution. 

Another large, structural aberration revealed by OGM was found in patient 4. A CNV gain on 8q in combination with a CNV loss comprising 8p12 next to a duplication SV (VAF 0.12–0.22). This duplication SV was inverted, repeating the segment, in proximity to the centromeric region. Taken together, these data strongly suggest the presence of additional 8q material corresponding to two 8q arms in conjunction with a duplication of 8p11 possibly based on the presence of an isodicentric chromosome 8q (Figure 1C). Karyotyping showed a normal result (46,XX [17]).

In patient 1, chromosome arms 7p and 9p were almost entirely affected by CN losses. Karyotyping result for this patient questioned a derivative chromosome consisting of 9q and 7q. While OGM is not capable of detecting whole-arm translocations because labeling does not cover the centromeric region, CNV losses of 7p and 9p were confirmed (VAF 0.45–0.50 with fractional CN ~1.0). Another gross CNV of about 23 Mbp in this patient was located on chromosome 10q, resulting in rearrangements within 10q (also indicated by several intrachromosomal fusion SVs) as well as translocations identifying chromosome 9p and 7p material on 10q that was not identified by karyotyping. VAF ranged from 0.44–0.53. 

The previous karyotyping result in patient 8 was 45,X,-Y [11]/46,XY [14], indicating the loss of chromosome Y. OGM revealed a large CN loss of about 22 Mbp on chromosome 20q as well as translocation SVs between chromosomes 20 and Y (VAF 0.07–0.21), redefining the karyotyping result as a rearrangement between chromosomes Y and 20. This OGM result adds additional structural information, suggesting that in fact chromosome 20q instead of Y material is lost. 

#### 3.2.2. Gene Fusions

Gene fusions reflect inversions, deletions, or translocations and are prevalent in a wide array of cancer types. In 9/11 patients, potentially relevant gene fusions were detected. Four, all of them B-ALL patients, harbored a t(9;22)(q34;q11) (patients 1, 2, 3, and 4) as detected by OGM. In all but one (karyotyping missed the translocation in patient 4), the t(9;22) was also detected by karyotyping and confirmed by RT-PCR. Using OGM, the breakpoint region can be narrowed down based on label positions. In patients 1 and 2, the breakpoints seem to be located in the minor BPR of *BCR.* This is in line with RT-PCR results, reporting the fusion product as an e1a2 (“minor”) transcript. In patients 3 and 4, the breakpoints are most likely located in the much smaller major BPR (6.850 bp compared to 71.558 bp of the minor transcript). Therefore, a precise localization within this region is more difficult based on OGM data. An RT-PCR performed for patient 3 detected a b3a2 (M-bcr) fusion transcript, which was confirmed by the OGM finding. An additional “minor transcript” was detected by RT-PCR that might be explained by alternative splicing [20]. 

**Figure 2:**
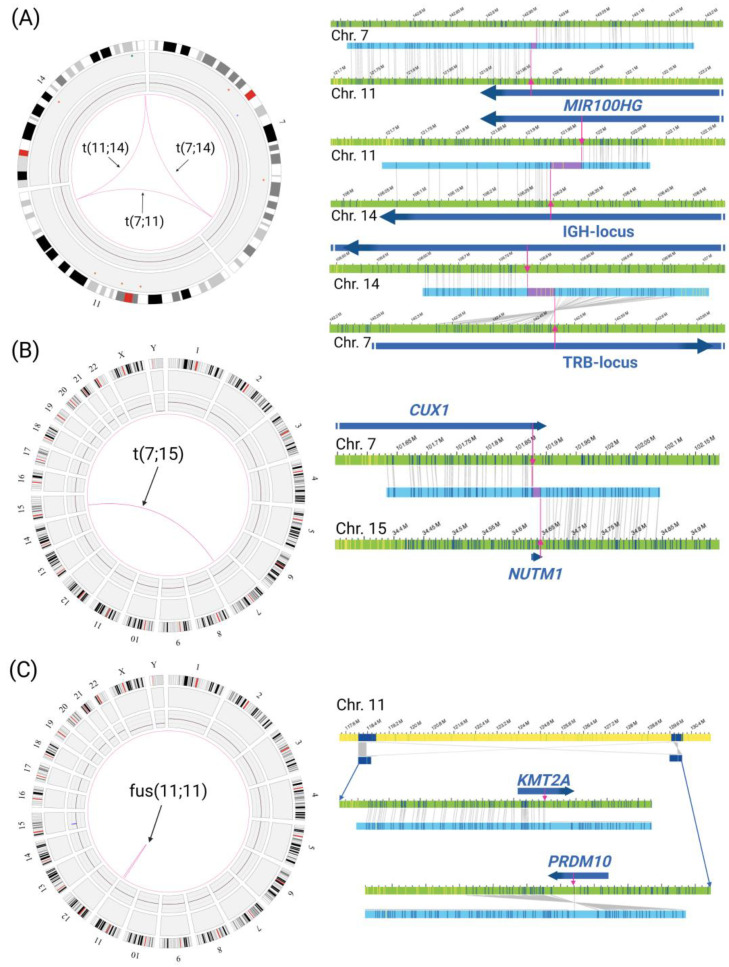
OGM detects rare or new gene fusions: Circos plots and respective SVs representing the gene fusions. Orientation of genes is indicated by arrows. (**A**) Three-way translocation involving the *IGH* locus on chromosome 14q, *TRB* locus on 7q, and *MIR100HG* micro-RNA on chromosome 11 (patient 5). (**B**) Circos plot and interchromosomal translocation SV reveal a t(7;15)(q22.1;q14) and the resulting *CUX1::NUTM1* fusion breakpoint region (patient 7). (**C**) Circos plot and intrachromosomal fusion SV showing *KMT2A* fused to *PRDM10* in patient 10. Created with BioRender.com.

In patient 5, a three-way translocation between the distal parts of chromosomes 7q, 11q, and 14q, affecting the *IGH* locus, *TRB* locus and a micro-RNA gene, was detected (Figure 2A). The *IGH* locus on 14q is disrupted twice and translocated partially to the *TRB* locus on 7q and to the long non-coding RNA gene *MIR100HG. MIR100HG* is disrupted a second time by a translocation between chromosomes 7q and 11q, but the breakpoint on chromosome 7q is located about 0.5 Mbp downstream of the *TRB* locus, towards the telomeric region. The translocation SVs had a relatively low VAF ranging from 0.06–0.10, thus corresponding to a small proportion of affected cells of about 5%. This three-way translocation t(7;11;14)(q34;q24.1,q32.33) could be detected by OGM as part of a complex rearrangement that was missed by standard diagnostics. Another translocation of *MIR100HG* (also known as *Mir-100-Let-7a-2-Mir-125b-1*) has already been described by Chapiro et al. [21]. In this study, the translocation t(11;14)(q24;q32) could be detected in two adult B-ALL patients using FISH and PCR. 

In patients 6 and 7, two previously reported but rare gene fusions were identified by OGM. Patient 6 showed a translocation t(12;22)(p13.31;q13.2) resulting in the gene fusion *EP300::ZNF384*. A *ZNF384* translocation was also detected by standard diagnostics, karyotyping and FISH analysis (Table S3), but the fusion partner remained unclear, possibly because there are currently 11 known fusion partners [22,23,24,25]. This missing result could be resolved by OGM. In addition, a clinically relevant t(7;15)(q22.1q14) resulting in a *CUX1::NUTM1*-fusion (Figure 2B) was found in patient 7, which was reported to have a normal karyotype (46,XX [25]).

In both T-ALL cases (patients 10 and 11), gene fusions were detected. Patient 11 exhibited a recurrent *TRA/D::LMO2* fusion [26]. Patient 10 showed an intrachromosomal inversion between 11q23.3 and 11q24.3, resulting in a *KMT2A::PRDM10* rearrangement (Figure 2C). While *KMT2A* rearrangements are associated with adverse risk in B-ALL, limited data are available for T-ALL [27]. The incidence is around 4–8% of T-ALL cases, preferentially with the partner gene *ENL* (*MLLT1*) [28]. The patient described here had a poor clinical course of disease, with ALL relapse and subsequent allogeneic hematopoietic stem cell transplantation. Recently, *PRDM10* has been reported as a potential *KMT2A* fusion partner [29].

#### 3.2.3. OGM Confirms Recurrent ALL Copy Number Alterations

**Figure 3:**
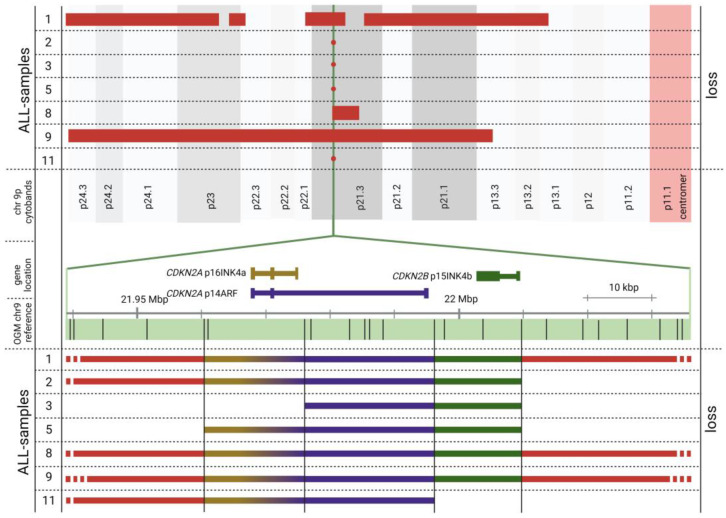
Estimated minimal region of overlap for *CDKN2A* and *CDKN2B* deletions on chromosome 9p (minus strand) based on OGM data. Large 9p deletions (**top**) and detailed view of 9p21.3 *CDKN2A* and *CDKN2B* genomic region (middle, bottom) are depicted. Top: Large losses of genomic material are shown as red bars, small deletions as red dots at the top. Detailed view of *CDKN2A* and *CDKN2B* genomic region includes the *CDKN2A* alternative reading frame and respective transcripts (*CDKN2A* transcripts p16INK4a and p14ARF and *CDKN2B* transcript p15INK4b). Colors of the deleted regions correspond to the affected transcripts. OGM label positions at breakpoint regions are vertically extended to the bottom of the figure. A minimal region of overlap can be hypothesized that affects the alternative reading frame of *CDKN2A* (p14ARF transcript) based on OGM data. Created with BioRender.com.

Our patients also revealed CN gains or losses in the investigated ALL-associated genes (Table S2 for gene overview and Table S3 for patient data). Most of these genes are reported to be involved in either lymphoid development (such as *PAX5* and *IKZF1*), lymphoid signaling, cell cycle and apoptosis regulation (mostly tumor suppressors such as *CDKN2A*), DNA repair, therapy response, or transcription factors [30,31]. Notably, 9p21 heterozygous deletions involving the genes *CDKN2A* and/or *CDKN2B* were detected in 7/11 patients (except for patient 1, who harbored a homozygous deletion). This is in line with previous results where *CDKN2A*/*2B* deletions were identified as the most common ones in ALL [32,33,34]. Figure 3 illustrates *CDKN2A/2B* deletions found by OGM: In 3 patients, large deletions (in the range of about 0.59 to 34.20 Mb) encompassed the *CDKN2A/B* genes. In 4 additional patients, rather small structural variants of approximately 24 to 95 kbp were found, unrevealing a putative region of minimum overlap of around 20 kbp for the 7 patients analyzed. Because of the different sizes of the deletions, these are not (easily) accessible using FISH and RT-PCR methodologies but could be easily accessed by OGM analysis.

Moreover, recurrent deletions of well-known genes associated with ALL were detected: aberrations of the transcription regulator *PAX5*, one of the most frequently affected genes in B-ALL [34,35], and *ETV6* were found in 4/11 samples. In patients 4 and 5 both genes, *PAX5* and *ETV6,* were deleted. *PAX5* exhibited an intragenic amplification in patient 9, and patient 6 had a homozygous deletion of *ETV6*. Another finding is a deletion at the *IGL* locus on chromosome 22q11.22 in 6/11 patients, involving the *VPREB1* gene in 4 of these patients. Antibody receptor gene loci are generally variable areas, but the pattern of the deletions detected in this study by OGM corresponds to the findings of Magnum et al. [36]. Deletions in this region are associated with a worse outcome in patients in conjunction with an *IKZF1* deletion [37]. As expected, also the IGH locus at 14q32 was affected in 3 patients and the TRA/D in 2 patients.

Some genes revealed a CN above 2 (normal diploid state for autosomes) because they were located on a chromosome that was affected by a trisomy or tetrasomy. For example, CN was increased for *TOX,* a nuclear factor and crucial regulator of the differentiation of tumor-specific T-cells, due to trisomy 8 in patients 3 and 4. In patients 5, 8, and 9, a deletion in close proximity to *TOX* was detected on chromosome 8.

Interestingly, patient 9 exhibited a translocation t(5;11)(q35.1;q24.3) with a VAF of about 0.19 that could not be related to known genes currently associated with ALL. Because of the possible implication of a translocation that might result in gene disruption, the region was analyzed in more detail: the *APLP2* gene on chromosome 11 is disrupted by this translocation, and the breakpoint on chromosome 5 also potentially results in a further gene disruption or novel gene fusion because the OGM output breakpoint region is located in or in close proximity to the *NPM1* gene.

**Figure 4:**
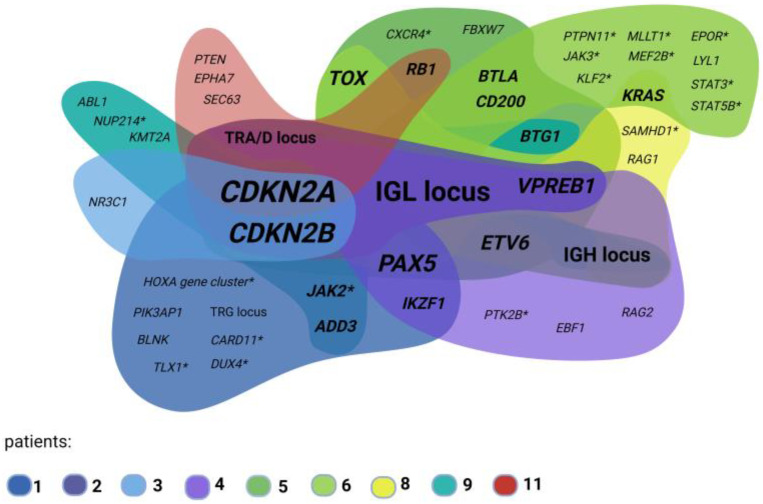
ALL-gene deletions/partial deletions overlap in the studied patient group. Single patients are visualized by unique color clouds. Gene cloud overlap shows frequency of affected genes: bigger font size corresponds to higher deletion frequency in the analyzed patient samples; core genes *CDKN2A/CDKN2B* are most frequently (partially) deleted and are visualized with the biggest font. Deletions in genes only detected in single patients show smallest font size. * Indicates ALL-associated genes with unknown effect of gene deletions. Legend describes patient numbers. Created with BioRender.com.

Overall, our study shows that OGM confirms karyotyping results and resolves recurrent aberrations. Interestingly, even in our small number of patients, there is a high overlap of affected genes on a genome-wide scale, as detected by OGM. Figure 4 shows detected deletions of known ALL genes individually for each patient and clearly highlights a large overlap of those in our small patient group.

## 4. Discussion

Standard karyotyping is often not sufficient to completely reveal the true nature of cancer cell karyotypes because of complex aberrations. OGM is capable of detecting gene fusions at a higher resolution compared to karyotyping without the necessity to preselect specific target genes to be analyzed by FISH or RT-PCR because multiple information can be acquired with only one methodology. Besides, as a whole genome approach, OGM can also detect relevant, novel SVs. In our patient group, five translocations were detected via classical karyotyping. Three of them were *BCR::ABL1* fusions, the most frequent fusion in adult ALL patients. Another was a t(12;22)(p13;q13), characterized by FISH as a *ZNF384* rearrangement. Using OGM, the fusion partner *EP300* could be identified. *EP300* is rarely found as a fusion partner and was estimated to occur in around 1% of pediatric patients by Gocho et al. [38]. Hirabayashi et al. (2017) discovered a distinct immune phenotype for *ZNF384*-related fusion genes, with low or negative expression for CD10 and aberrant expression of CD13 and/or CD33 [25]. However, *EP300::ZNF384*-associated loss of CD10 expression is in line with our patients’ results in flow cytometry. *EP300::ZNF384* fusions show a better response to steroid monotherapy as well as conventional chemotherapy and present with no significant elevation of WBC, while *ZNF384*-fusions with a different partner gene, *TCF3*, result in an elevated WBC level and poor response to prednisolone and conventional chemotherapy [25,38]. Thus, OGM is not only a useful tool for the detection of rare and unknown fusion partners but also opens the possibility of individualizing therapy.

### 4.1. OGM Can Detect Rare Gross Structural Rearrangements

A ring chromosome 9 could be redefined in one patient by manual inspection of the data, outperforming karyotyping and FISH. Similarly, the presence of an idic(8q) is very likely based on OGM data in one patient, representing another putative aberration not detected in the initial chromosome analysis. The missed detection of an idic could be due to culture artifacts because repeated cell divisions might have resulted in the loss of the isochromosome, as reported before [39]. In contrast to cell cultivation following karyotyping and RT-PCR, OGM analysis uses DNA material without prior cultivation or amplification steps and therefore directly mirrors the genetic composition of the sample, which makes its presence very likely, although this cannot be proven by our results. Our results support the idea that OGM might add structural genetic information because isochromosomes might be detectable in certain cases by this methodology, despite the limitation of OGM to detect whole-arm translocations. This, of course, depends on the position of the breakpoints and only works for breakpoints outside the centromeric region. In patient 2, an additional, atypical Philadelphia chromosome was detected by OGM without the need for detailed follow-up analysis. Even in our small patient group, these results clearly show that the OGM method is not only able to detect translocations that usually cannot be detected by molecular methods. It also reveals even more complex major structural changes with a higher resolution than karyotyping.

### 4.2. Rare Gene Fusions

OGM can also characterize rare gene fusions that are not likely to be examined in a standard routine diagnostic workup. In patient 7, OGM revealed a *CUX1::NUTM1* fusion that was absent in karyotyping. A *NUTM1* rearrangement was previously reported in childhood ALL only, with a prevalence ranging from 3–5% in infants to <1% in children [10,40]. The patient with a *CUX1::NUTM1* fusion in our group was a 35-year-old female; to the best of our knowledge, this was the first adult case reported with this aberration. *NUTM1*-rearrangements occur with different fusion partners. *CUX1::NUTM1* fusions are associated with an upregulation of HOXA cluster genes [41,42] that are suspected to play a role in leukemic hematopoiesis, as their aberrant expression is assumed to have a leukemogenic effect. A study in mice linked overexpression of *HOXA10* to AML and impairment of B-cell development [43]. *NUTM1*-rearrangements often seem to occur without additional recurrent known deletions (such as *CDKN2A/B, ETV6, PAX5,* and *IKZF1)* [40], and hence can be suspected as oncogenic drivers [42]. In line with this, our patient did not reveal any additional ALL-specific aberrations except for a partial deletion in the *CUX1* gene with uncertain significance. *NUTM1*-rearrangements seem to have a favorable prognosis [40,42], which correlates with the clinical course of the patient described here. OGM thus showed a rare gene fusion in a cytogenetically normal patient, potentially unraveling the oncogenic driver and adding information to the patient’s prognosis.

Besides the overall approach focusing on ALL-related genes, an additional translocation was revealed by OGM and analyzed by manual inspection, affecting the genes *APLP2* and potentially *NPM1*. *APLP2* is a member of the Alzheimer precursor protein family. Another member of this family, amyloid precursor protein, encoded by *APP*, was previously reported to be a clinically significant and prognostic factor in AML1-ETO cases [44]. Moreover, *APP* and APP-like protein-2 (*APLP2*) are deregulated in cancer cells and linked to increased tumor cell proliferation, migration, and invasion [45]. *NPM1* represents the most frequently mutated gene in AML, is known as a good prognostic marker, and thus might be of potential clinical significance and targeted therapy [46] in this patient. Further analyses should be conducted to analyze the underlying structural change in molecular detail.

### 4.3. CDKN2A/2B Loss

Several recurrent abnormalities that were reported in ALL could be found in our patients. The most common were deletions spanning the *CDKN2A/CDKN2B* locus on 9p21.3, deleting these genes at least partially. Both genes encode proteins involved in cell cycle regulation; p16INK4a (encoded by *CDKN2A)* and p15INK4b (encoded by *CDKN2B). CDKN2A* has an alternative first exon, encoding the p14ARF protein (alternative reading frame), which is involved in the p53 pathway and important for cell cycle regulation. Gonzalez-Gil et al. analyzed several studies covering *CDKN2A/CDKN2B* deletion frequencies in adult and childhood B- and T-ALL [33]. Karyotyping revealed 9p deletions with frequencies ranging from 7% to 20% for del(9p) or abnormal 9p in adult patient groups. Using methods enabling higher resolution at the single-gene level, such as MLPA or FISH, the numbers were higher (24% to 50% with MLPA and 24% to 43% with FISH) [33]. OGM expands these previous data and enables a detailed view of the structural basis of these deletions. Altogether, 7 patients (64%) exhibited a *CDKN2A* deletion, and 6 (55%) of those patients had an additional *CDKN2B* deletion, confirming the high proportion of affected patients by this aberration. Moreover, OGM revealed the heterogeneity of *CDKN2A/2B* deletions, the result of rather small deletions or large losses of chromosome 9p material. Follow-up investigations on *CDKN2A/2B* deletions are of great interest, as there is evidence that they are prognostic factors in adult ALL patients. In adults, there is evidence that *CDKN2A/2B* deletions are associated with a poor prognosis [47,48,49]. These findings, as well as the high frequency with which *CDKN2A/2B* deletions occur, promote further investigation and the consideration of including *CDKN2A/2B* deletions in future risk assessments, especially in adult patients. Those are even more interesting as there are possible targeted treatment options for *CDKN2A/2B* deletions. CDK4/6 inhibitors, which are capable of substituting for the enzyme, are already used for breast cancer treatment. Experiments treating T- and B-ALL xenografts with Palbociclib and Ribociclib suggest a possible effect on CDK4/6 inhibitors, and their application in ALL patients could be a therapeutic option in the future, which remains to be studied [50].

### 4.4. Limitations

As already outlined in previous studies [15,16,51], OGM also has its limitations, which affected our ALL-gene panel analysis: The *CRLF2* (Cytokine Receptor Like Factor 2) gene is involved in signaling pathways of hematopoiesis; aberrations in this gene belong to the ALL subclass BCR-ABL1-like [7]. It is located on chromosome X or Y in the PAR region. Smith et al. (2022) [15] were able to detect an interstitial deletion on chromosome X with a resulting *CRLF2::P2RY8* fusion using OGM de novo analysis and the hg38 reference. Lestringant et al. (2021) [16] failed to detect a *CRLF2::P2RY8* fusion (caused by an interstitial deletion) with RVP analysis and the hg19 reference. The authors outline that the missed aberrations might be due to a low VAF, low coverage of some regions that can potentially be caused by reference errors or other calls filtered out due to high inter-individual variability or highly repeated sequences such as centromeres, telomeres, and PARs. In these regions, it is difficult to study recurrent aberrations, such as *CRLF2* rearrangements or SVs in *NOTCH2* (partially masked) by OGM, which holds also true for the results of our study.

## 5. Conclusions

In adult ALL patients, genetic aberrations are currently under-explored and can be investigated more extensively using OGM than in standard routine diagnostics, as revealed by the number of ALL-related aberrations in the presented data. Interestingly, a genetic variety in conjunction with overlapping affected genes was found in previous studies and could also be mirrored in our small patient group. We were able to show that the OGM offers many advantages in clinical routine diagnostics of adult ALL patients as it detects multiple aberrations in one single workflow. Not only can structural alterations such as ring chromosomes, a Philadelphia-like chromosome, or putative isodicentric chromosome be verified, but the results can also be used for the diagnosis of ALL in adults, enabling accurate treatment as well as a significantly increased detection rate and resolution compared to karyotyping and FISH.

## Figures and Tables

**Table 1:** Patient characteristics. f: female, m: male; ^1^ bone marrow aspirate, ^2^ peripheral blood, ^3^ BM infiltration (Histology) or blastcount in PB (cytology), ^4^ not otherwise specified.

Patient ID	Age	Sex	Sample Type	Diagnosis	Initial Diagnosis	GMALL Risk	WHO 2022 Classification	Disease Stage	Blasts ^3^
1	18	m	BM ^1^	Common B-ALL	07/21	High	B-ALL with *BCR::ABL1*	Initial	>90%
2	60	f	BM ^1^	Common B-ALL	02/22	High	B-ALL with *BCR::ABL1*	Initial	>90%
3	43	f	BM ^1^	Common B-ALL	08/21	High	B-ALL with *BCR::ABL1*	Progress	>90%
4	62	f	BM ^1^	Common B-ALL	05/22	High	B-ALL with *BCR::ABL1*	Initial	80%
5	34	m	BM ^1^	Common B-ALL	04/21	High	B-ALL, NOS ^4^	Initial	85%
6	18	m	BM ^1^	Pro B-ALL	05/21	High	B-ALL, NOS ^4^	Initial	40%
7	35	f	PB ^2^	Pre B-ALL	04/21	High	B-ALL, NOS ^4^	Initial	>90%
8	58	m	BM ^1^	Pro B-ALL	03/21	High	B-ALL, NOS ^4^	Refractory	80%
9	26	m	BM ^1^	Common B-ALL	01/22	Standard	B-ALL, NOS ^4^	Initial	>90%
10	22	m	BM ^1^	Thymic T-ALL	06/19	Standard	T-ALL, NOS ^4^	Progress	<5%
11	33	f	BM ^1^	Thymic T-ALL	08/22	Standard	T-ALL, NOS ^4^	Initial	>90%

## Data Availability

The data presented in this study are openly available at https://zenodo.org (accessed on 22 December 2022) [DOI: 10.5281/zenodo.7291345]. OGM raw data sets and other data that support the findings of the study are available from the corresponding author upon request.

## Supplementary Materials

The following supporting information can be downloaded at: https://www.mdpi.com/article/10.3390/genes14030686/s1, Table S1: OGM quality parameter; Table S2: bed file information document; Table S3: detailed results; File S1: bed file ALL genes.
