# Supplementary material for: Optical Genome Mapping Reveals and Characterizes Recurrent Aberrations and New Fusion Genes in Adult ALL
Source: Genes (Basel). 2023 Mar 9;14(3):686. doi: 10.3390/genes14030686 (PMC10048194; doi:10.3390/genes14030686)
Supplement: Supplementary file 1 [file genes-14-00686-s001.zip › genes-2153564-supplementary/Supplementary documents/Table S2.pdf]

Supplementary document 2 information

ALL-gene .bed file information

The .bed file contains genes associated with ALL and lymphoid malignancies (according to GMALL study group and Swerdlow et al., WHO Classification of Tumors of Hematopoietic and Lymphoid Tissues, *IARC*, 2022) and genes that are recurrently identified in ALL cases and considered potentially relevant for ALL according to current literature. The genomic position of the below mentioned genes was verified for hg19 gene transcripts using “Entrez Gene” by the National Library of Medicine (https://www.ncbi.nlm.nih.gov/gene/). The .bed file includes genes associated with B-ALL and T-ALL as well as breakpoint regions.

1	164528421	164821068	PBX1
1	47681962	47698007	TAL1
1	87794563	87814606	LMO4
1	47715811	47779819	STIL
1	115247090	115259392	NRAS
1	179068465	179198812	ABL2
1	27022506	27108595	ARID1A
1	23884417	23885992	ID3
1	65298912	65533429	JAK1
1	156433519	156470567	MEF2D
1	120454176	120612276	NOTCH2
2	136871919	136875719	CXCR4
2	198254508	198299817	SF3B1
2	61704984	61765491	XPO1
3	112182813	112218319	BTLA
3	112051420	112081659	CD200
3	38180152	38184513	MYD88
4	153241696	153457244	FBXW7
4	191005267	191007077	DUX4
4	55095460	55164412	PDGFRA
4	106067032	106200960	TET2
5	131396348	131398897	IL3
5	158122928	158526924	EBF1
5	142657496	142815077	NR3C1
5	170736252	170739143	TLX3
5	49432858	149492928	CSF1R
5	35856993	35879705	IL7R
5	149493402	149535408	PDGFRB
5	1253282	1295183	TERT
6	93949738	94129277	EPHA7
6	135502446	135540310	MYB
6	44225903	44233288	NFKBIE
6	108188960	108279412	SEC63

6	138188396	138204449	TNFAIP3
7	50344312	50472798	IKZF1
7	141998851	143510972	TRB locus
7	38279625	38407656	TRG locus
7	27132614	27239710	HOXA-gene-cluster
7	140413128	140624729	BRAF
7	2945776	3083501	CARD11
7	148504475	148581383	EZH2
7	124462440	124569879	POT1
8	59717971	60031767	TOX
8	42128820	42189978	IKBKB
8	128748477	128755197	MYC
8	27168995	27316908	PTK2B
8	103264501	103424928	UBR5
9	133710641	133763062	ABL1
9	133531000	133730187	breakpoint region BCR::ABL1
9	133589842	133710453	most common breakpoint region BCR::ABL1
9	21967751	21975132	CDKN2A p16INK4a
9	21967751	21994490	CDKN2A p14ARF
9	22002902	22009304	CDKN2B
9	36833266	37034265	PAX5
9	108424738	108425403	TAL2
9	139388885	139440500	NOTCH1
9	4985245	5128183	JAK2
9	134000973	134110050	NUP214
10	102891057	102897546	TLX1 (HOX11)
10	21823101	22032559	MLLT10
10	89623382	89731687	PTEN
10	97948927	98031326	BLNK
10	98353072	98480271	PIK3AP1
10	111756131	111895323	ADD3
10	64571756	64578927	EGR2
11	118307207	118397547	KMT2A
11	8245856	8285425	LMO1 (RBTN1)
11	33880123	33913623	LMO2 (RBTN2)
11	85668219	85780126	PICALM
11	36531903	36601312	RAG1
11	36613493	36619786	RAG2
11	108093794	108239829	ATM
11	102188215	102210134	BIRC3
11	69455924	69469242	CCND1/BCL1
11	85955795	85989852	EED
11	532242	535576	HRAS
11	32409321	32457085	WT1
12	11802608	12048311	ETV6
12	6775643	6798738	ZNF384

12	25358180	25403863	KRAS
12	92534054	92539622	BTG1
12	112856751	112947722	PTPN11
13	48877883	49056026	RB1
13	28577411	28674713	FLT3
13	41129804	41240778	FOXO1
14	106053226	107288019	IGH locus
14	22090057	23021075	TRA/D locus
14	96176284	96180462	TCL1
14	103243786	103377837	TRAF3
15	34635516	34649936	NUTM1
15	90626277	90645700	IDH2
15	66679250	66783882	MAP2K1
15	88402982	88799970	NTRK3
16	3775055	3930714	CREBBP
16	81812896	81996290	PLCG2
17	7571720	7590868	TP53
17	62006100	62009691	CD79B
17	53342321	53402548	HLF
17	29421945	29704695	NF1
17	40465342	40540460	STAT3
17	40351195	40428409	STAT5B
17	30264026	30328057	SUZ12
18	52889416	53255423	E2-2/TCF4 Deletion
18	60790579	60987002	BCL2
18	52889416	53255423	TCF4
19	1609291	1652614	TCF3
19	13209847	13213672	LYL1
19	11487883	11494990	EPOR
19	17935591	17958791	JAK3
19	16435637	16439496	KLF2
19	35783064	35804710	MAG
19	19256376	19281072	MEF2B
19	6210392	6279986	MLLT1
19	1438395	1440494	RPS15
19	1609291	1652614	TCF3
19	56166401	56186082	U2AF2
20	35518176	35580111	SAMHD1
21	36160098	36421595	RUNX1 - critical region for iAMP21
22	22380474	23265085	IGL locus
22	22599087	22599927	VPREB1
22	23522696	23660224	BCR
22	23630360	23637210	M-bcr breakpoint region
22	23524427	23595985	m-bcr breakpoint region
22	23613780	23615267	v-bcr breakpoint region
22	23654024	23655073	my-bcr breakpoint region
22	41488596	41576081	EP300

X	100604438	100645784	BTK
X	1314869	1331542	CRLF2 on X
X	44732434	44972024	KDM6A/UTX
X	1581474	1656078	P2RY8 on X
X	133507342	133562820	PHF6
Y	1264869	1281542	CRLF2 on Y
Y	1531474	1606078	P2RY8 on Y
